# Depression of presynaptic excitation by the activation of vanilloid receptor 1 in the rat spinal dorsal horn revealed by optical imaging

**DOI:** 10.1186/1744-8069-2-8

**Published:** 2006-02-17

**Authors:** Kei Kusudo, Hiroshi Ikeda, Kazuyuki Murase

**Affiliations:** 1 University of Fukui, 3-9-1 Bunkyo, Fukui 910-8507, Japan

## Abstract

In this study, we show that capsaicin (CAP) depresses primary afferent fiber terminal excitability by acting on vanilloid receptor 1 (TRPV1 channels) of primary afferent fibers in adenosine 5'-triphosphate (ATP)- and temperature-dependent manner using two optical imaging methods. First, transverse slices of spinal cord were stained with a voltage-sensitive dye and the net excitation in the spinal dorsal horn was recorded. Prolonged treatment (>20 min) with the TRPV1 channel agonist, CAP, resulted in a long-lasting inhibition of the net excitation evoked by single-pulse stimulation of C fiber-activating strength. A shorter application of CAP inhibited the excitation in a concentration-dependent manner and the inhibition was reversed within several minutes. This inhibition was Ca^++^-dependent, was antagonized by the TRPV1 channel antagonist, capsazepine (CPZ), and the P_2_X and P_2_Y antagonist, suramin, and was facilitated by the P_2_Y agonist, uridine 5'-triphosphate (UTP). The inhibition of excitation was unaffected by bicuculline and strychnine, antagonists of GABA_A _and glycine receptors, respectively. Raising the perfusate temperature to 39°C from 27°C inhibited the excitation (-3%/°C). This depressant effect was antagonized by CPZ and suramin, but not by the P_2_X antagonist, 2', 3'-O-(2,4,6-trinitrophenyl) adenosine 5'-triphosphate (TNP-ATP). Second, in order to record the presynaptic excitation exclusively, we stained the primary afferent fibers anterogradely from the dorsal root. CAP application and a temperature increase from 27°C to 33°C depressed the presynaptic excitation, and CPZ antagonized these effects. Thus, this study showed that presynaptic excitability is modulated by CAP, temperature, and ATP under physiological conditions, and explains the reported central actions of CAP. These results may have clinical importance, especially for the control of pain.

## Background

Yaksh et al. [1] first reported that an intrathecal injection of capsaicin (CAP) depletes substance P from primary sensory neurons and causes a prolonged increase in the thermal and chemical, but not mechanical, pain thresholds. Since then, a number of studies have shown that CAP produces analgesic action at the spinal level [2-4]. However, as reported by Nagy et al. [5], the depletion of spinal cord substance P by CAP may not be sufficient to explain the changes induced by CAP.

Recent studies in peripheral axons revealed that CAP acts on vanilloid receptors 1 (TRPV1 channels) that are present on fine afferent fibers [6]. The receptor channels are activated by painful heat stimuli (>43°C), as well as noxious chemical stimuli [6,7]. The flow of Ca^++ ^and Na^+ ^through the channels induces depolarizing receptor potentials at the peripheral terminals, which generate action potentials that transmit pain information [6]. Following such primary excitatory actions, treatment with CAP often produces secondary inhibitory actions. The application of TRPV1 channel agonists at afferent fiber bundles blocks conduction of the action potential [8-11]. Adenosine triphosphate (ATP) also affects TRPV1 channels indirectly through the activation of G-protein, and sensitizes them so as to be activated at a lower temperature [7]. In the superficial layers (laminae I-II) of the dorsal horn, TRPV1 channels are abundant, especially at presynaptic elements of unmyelinated C-fiber origins [12]. However, their roles in afferent signal transmission are unclear.

We have been recording neuronal excitation in central slices by using an optical method with a voltage-sensitive dye [13,14], and investigated the neuronal circuitry and plasticity in spinal cord slices [15,16]. Recently, we succeeded in staining primary afferent fibers anterogradely from the dorsal root, and optically recorded the presynaptic excitation exclusively [17]. Utilizing these two types of optical imaging methods, the visualization of presynaptic excitation and net excitation of pre- and postsynaptic elements, we investigated the mechanism of CAP action in the spinal cord. In addition, we examined the temperature dependence of CAP activity, because, unlike other brain regions such as the olfactory bulb and the hippocampus, we noticed that the magnitude of optical signal, especially in the superficial dorsal horn, changes depending on the perfusate temperature. Further, we speculated that TRPV1 channels contribute to this temperature dependence. In this study, we report that CAP acts on TRPV1 channels, most likely at presynaptic elements, that are consistently sensitized via P2Y receptors, and, therefore, that CAP can inhibit the afferent signal transmission even at physiological temperature.

## Results

### The effects of CAP on net excitation of pre- and postsynaptic elements

**Figure 1:**
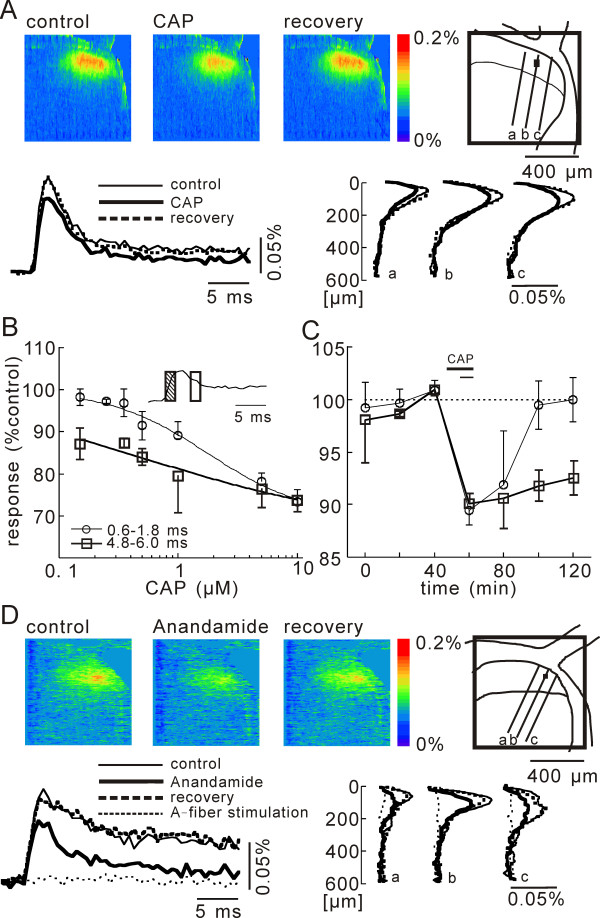
**Depression of afferent-evoked net neuronal excitation by CAP**. **A**) A pseudo-color image sequence of stimulation-induced net optical responses measured from a slice bathed in a control solution (left), a CAP (0.5 μM)-containing solution (center), and the control solution for recovery (right). At left bottom, superimposed time traces obtained at the black square in the schematic drawing are illustrated for the respective images. At right bottom, the spatial distributions along the dorso-ventral lines in the schematic drawings are also illustrated. **B**) Concentration-dependent inhibition of net neuronal excitation by CAP at the period of 0.6–1.8 ms in evoked response (circles), and of 4.8–6.0 ms (squares). The curves were drawn according to the equation *I *= *I*_*max*_/[*1*+(*IC*_50_/[*CAP*])^*N*^] (IC_50 _= 0.5 μM & I_max _= -30% N = 1.0 at 0.6–1.8 ms of evoked response, and IC_50 _= 1.7 μM & I_max _= -33% N = 0.5 at 4.8–6.0 ms of evoked response) **C**) Changes in normalized time courses of the magnitudes of net neuronal excitation by CAP application with two different durations, 10 and 20 min (circles and squares, respectively). The period of CAP application is indicated by the bar. **D**) Pseudo-color image sequence of stimulation-induced net optical responses measured from a slice bathed in a control solution (left), a anandamide (50 μM)-containing solution (center), and the control solution for recovery (right). At left bottom, superimposed time traces obtained at the black square in the schematic drawing are illustrated for the respective images and at A-fiber stimulation. At right bottom, the spatial distributions along the dorso-ventral lines a, b and c in the schematic drawings are also illustrated. Excitation evoked by the dorsal root stimulation of A-fiber-activating strength in the normal condition is illustrated with dotted lines in the time traces and the spatial distributions. No detectable changes were observed.

An example of optically recorded neuronal activity elicited by the stimulation is shown in Figure 1A. In a transverse slice of the lumbar spinal cord stained with the voltage-sensitive dye, stimulation was applied to one of the dorsal roots attached to the slice, and neuronal activity in the dorsal horn region ipsilateral to the stimulated dorsal root was recorded optically. Single-pulse dorsal-root stimulation of C fiber-activating strength evoked prolonged (> 100 ms) neuronal activity in the superficial dorsal horn (Fig. 1A). Stimulation of A-fiber-activating strength elicited no detectable response in the region (Fig. 1D) [14]. Previously. we concluded that the optical response mostly reflects long-lasting neuronal activities of C-fiber origin, such as a burst of action-potential firings, synaptic bombardment and an increase in membrane excitability, which are known to be present after intense dorsal root stimulation [14,15]. Note that the optical response is composed of both excitation of afferent fibers and terminals and the following postsynaptic excitations.

**Figure 2:**
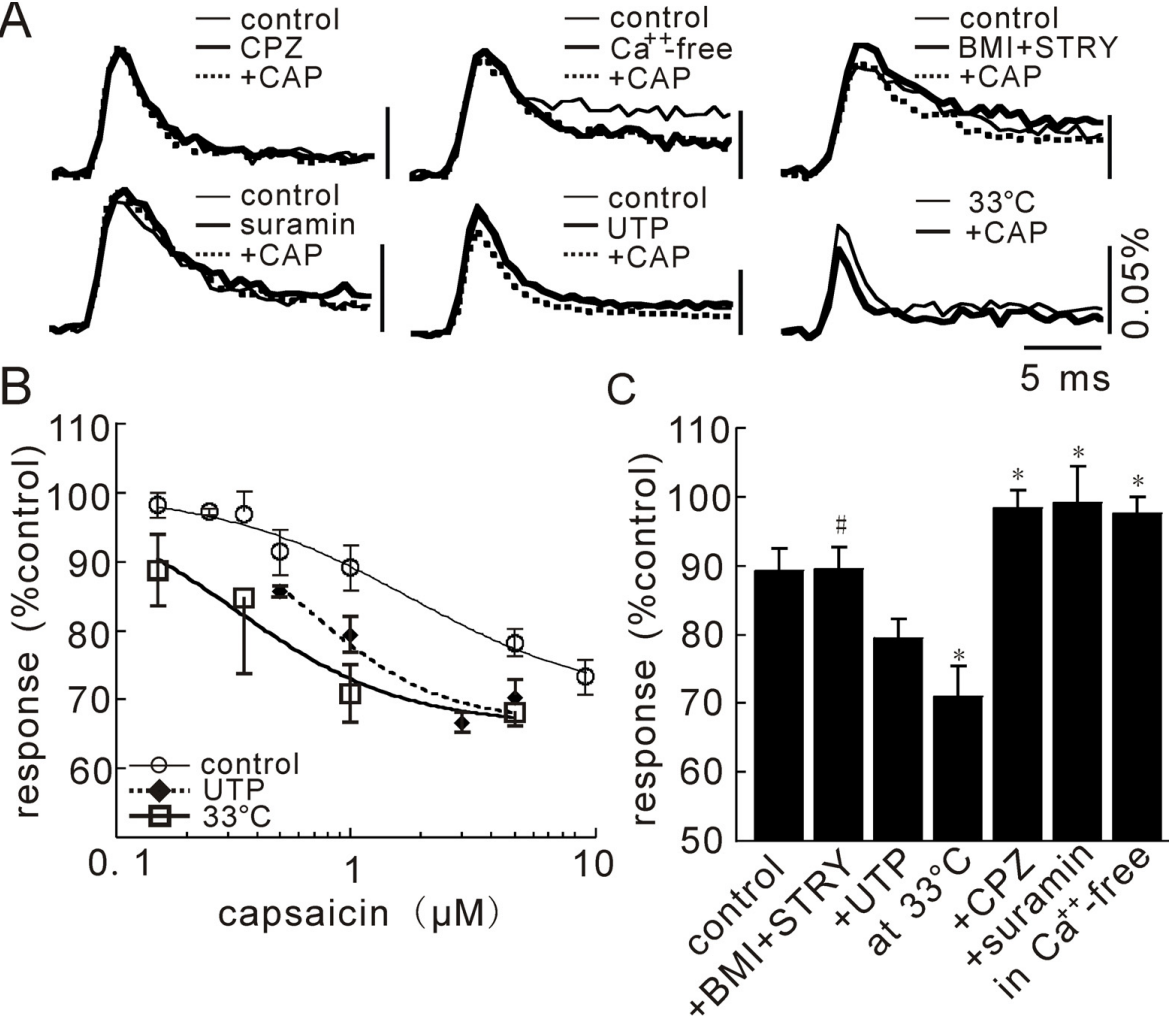
**Pharmacology of CAP-induced depression of net neuronal excitation**. **A**) Time traces obtained using various agents. The effect of CAP was antagonized by the vanilloid receptor 1 antagonist, CPZ (20 μM), suramin (100 μM), and a Ca^++^-free medium. It was not affected by BMI (10 μM) and STRY (3 μM), and was potentiated by UTP (30 μM) and at a higher temperature of 33°C. **B**) Concentration dependencies of CAP effect in normal solution at 27°C (circles) and 33°C (squares), and in UTP at 27°C (diamonds). Points were fitted with IC_50 _= 1.7 μM, Imax = -30% and N = 1.0 for 27°C, IC_50 _= 0.3 μM, Imax = -34% and N = 1.2 for 33°C, and IC_50 _= 0.6 μM, Imax = -33% and N = 1.5 for UTP. **C**) Averaged percent control response in various agents in 4–6 slices. Statistical significance and insignificance to control change are indicated with signs, * (p < 0.05) and # (p > 0.5), respectively. Although +UTP was not significant, it shifted the dose-response curve reasonably well (diamonds in C).

First, we investigated the action of CAP on the net neuronal excitation consisting of pre- and postsynaptic components. At a perfusate temperature of 27°C, the bath application of CAP for 10 min inhibited the neuronal excitation most effectively at the superficial regions of the dorsal horn, as shown in Figure 1A. The inhibitory effect was concentration-dependent (Fig. 1B). The effect was less during the initial several milliseconds of elicited excitation than during the delayed part (IC_50 _= 0.5 μM, Imax = -30%, N = 1.0 for the initial 0.6–1.8 ms, v.s. IC_50 _= 1.7 μM, Imax = -33%, N = 0.5 for later 4.8–6.0 ms). As shown in Figure 2A, the excitation in the former period was unchanged in a Ca^++^-free condition, and, thus, reflects predominately the excitation of presynaptic elements, and the latter reflects the following postsynaptic excitation (see also Figures 4A and 4D and Figure 6B for the duration of presynaptic excitation). In the following results pertain to the initial period unless otherwise noted. It is interesting to note that the depressant effect lasted over an hour after a prolonged application of CAP of over 20 min (Fig. 1C). In the following experiments, we used a 10 minutes application of 1 μM CAP, because the effect (-11.8 ± 1.4%, n = 3, p = 0.02) was reversible within several minutes. The second application of CAP at one hour after recovery from the first application produced a similar inhibition in two slices, but not in three slices (data not shown). The natural ligand of the TRPV1 channel, anandamide (50 μM, 10 min), also depressed the neuronal excitation in the superficial laminae (-23.3 ± 4.43%, n = 3, p = 0.03) (Fig. 1D). Although the 10–23% change in excitation may seem small, we believe that it is not marginal since it is caused by the change in the magnitude or in the number of evoked action potentials (see the Discussion for details).

**Figure 3:**
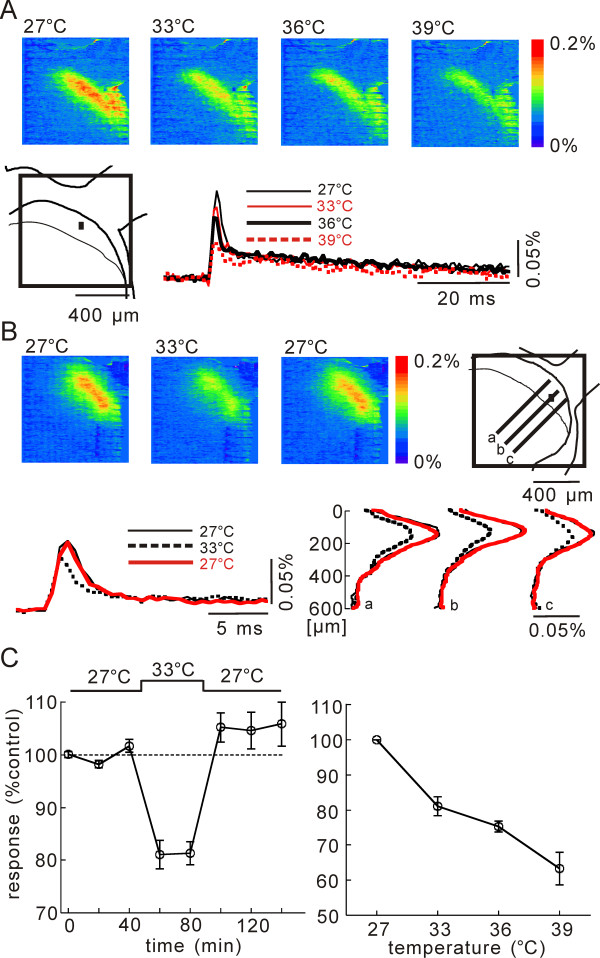
**Effect of temperature increase on C-fiber evoked net excitation**. **A**) Pseudo-color image sequence of stimulation-induced optical responses measured in a slice in perfusion solution at 27°C, 33°C, 36°C, and 39°C (from left to right). Superimposed time traces of optical signals obtained at 27°C (thin black), 33°C (thin red), 36°C (bold black), and 39°C (dashed red). These records were obtained by spatial average of the optical signals of 8 × 8 pixels corresponding to the black square area (50 × 50 μm) shown in the schematic drawing of the dorsal horn. Dotted line in the optical signal records indicates the baseline level. **B**) Pseudo-color image sequence of stimulation-induced optical responses measured from slices bathed in 27°C, 33°C, and 27°C (form left to right). Superimposed time traces obtained at 27°C (thin black), 33°C (dashed black), and 27°C (red) are indicated at left below. Spatial distributions of the magnitude of excitation along lines in schematic drawing are illustrated at right below. **C**) Normalized time course of magnitude of optical signals showing the averaged effect induced by a change of temperature from 27°C to 33°C (left), and the averaged temperature-dependency of depression (right), in 4 slices.

**Figure 4:**
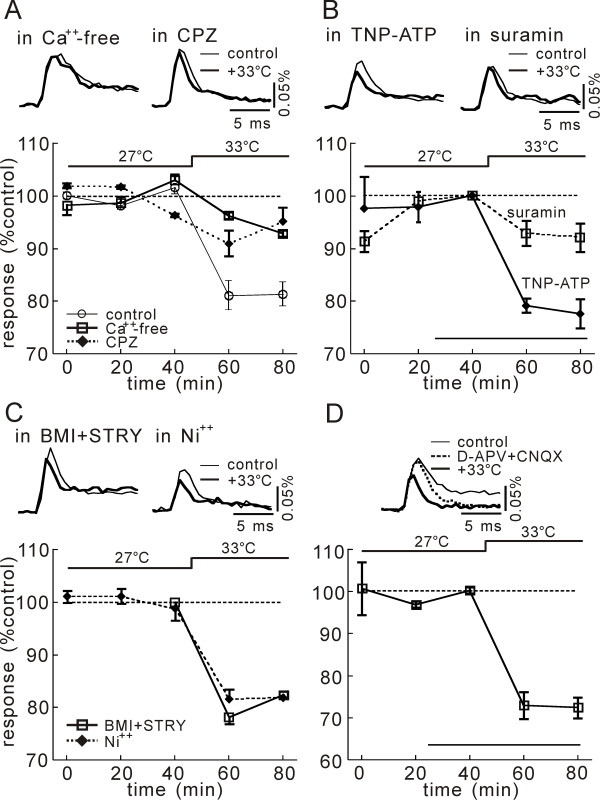
**Pharmacology of temperature effect on net excitation**. **A**) Antagonism by CPZ (20 μM) and a Ca^++^-free medium. Upper inserts show the time traces of optical signals at 27°C (thin) and 33°C (bold). Bottom graph shows the averaged changes in normalized magnitude of optical signal obtained in 4 slices when temperature was altered from 27°C to 33°C as indicated by the bar above. Circles, squares and diamonds indicate results obtained in the normal solution (control), in a Ca^++^-free medium, and CPZ-containing solution, respectively. **B**) Antagonism by suramin (100 μM) and potentiation by TNP-ATP (1 μM). Bottom line in the graph indicates the duration of drug application. **C**) No effect of BMI (10 μM) and STRY (3 μM) (squares), and Ni^++ ^(diamonds) is observed on temperature effect. **D**) Augmented effect in APV (50 μM) and CNQX (10 μM).

**Figure 5:**
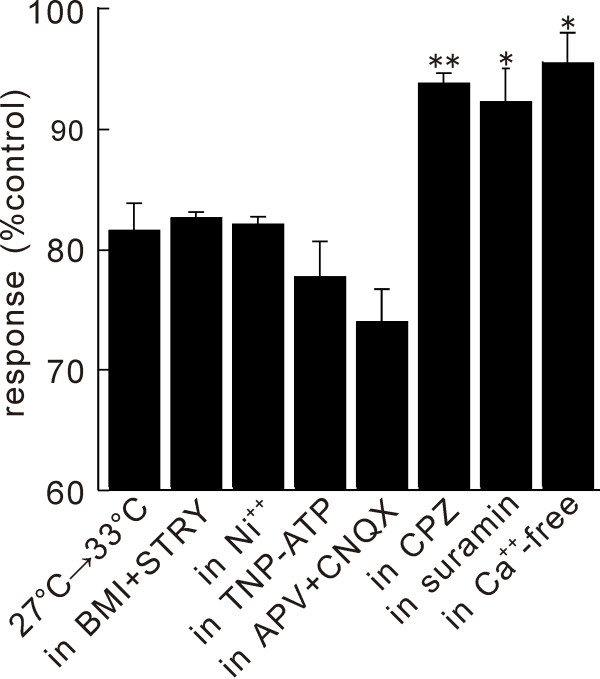
**Changes of net excitation induced by temperature increase from 27°C to 33°C in various conditions**. Each bar exhibits the averaged results obtained in 4–7 slices. Statistical significance to control change is indicated with signs, ** (p < 0.01) and * (p < 0.05), respectively.

**Figure 6:**
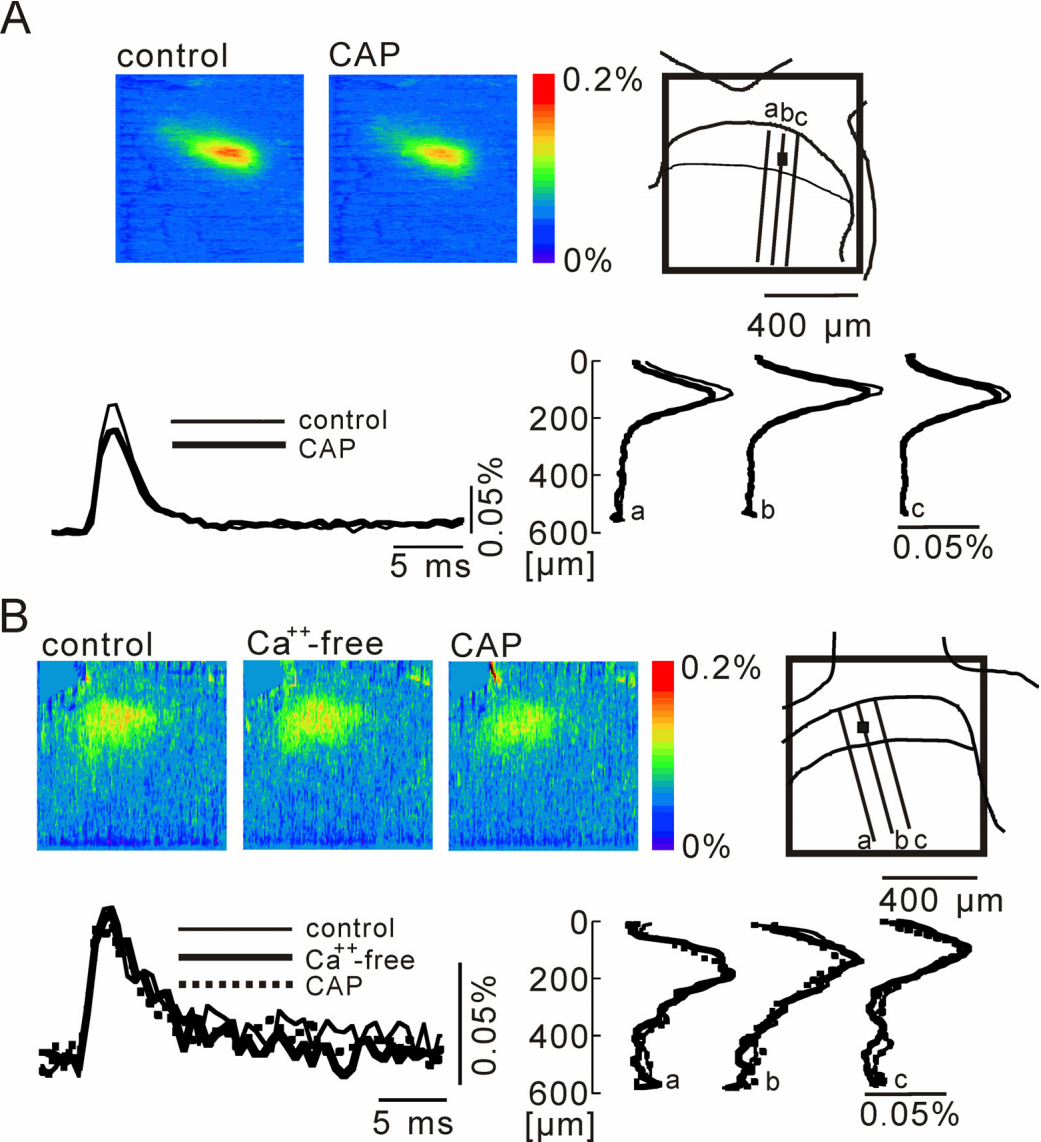
**Depression of presynaptic excitation by CAP**. **A**) Pseudo-color image sequence of stimulation-induced presynaptic excitation measured from a slice in the control solution (left) and with CAP (right) at 27°C. Superimposed time traces at the black square in the schematic drawing, as well as spatial distributions along the dorso-ventral lines a, b and c, are illustrated at bottom. **B**) No effect of CAP in a Ca^++^-free condition.

In slices from rats lacking most of the CAP sensitive afferent fibers by neonatal CAP treatment, the bath application of CAP did not inhibit the neuronal excitation (+4.3 ± 5.8%, n = 4). Therefore, the inhibition of neuronal excitation by CAP is likely due to the change in excitability of capsaicin sensitive afferent fibers.

The pharmacology of the CAP action is shown in Figure 2. The inhibition by CAP was not observed in the presence of the TRPV1 channels antagonist, capsazepine [18] (CPZ, 20 μM, applied for 40 min from 20 min before CAP application), or in the absence of Ca^++^, which is the major ion carried through TRPV1 channels [6]. The possibility that the depression might be due to the activation of inhibitory interneurons by CAP was excluded, since the CAP action was unaffected by the presence of bicuculline (BMI, 10 μM) and strychnine (STRY, 5 μM), antagonists of GABA_A _and glycine receptors, respectively. However, a possible contribution of interneurons that contain other inhibitory transmitters still remained. In the presence of CPZ, anandamide depressed the neuronal excitation by -10.8 ± 1.7% (n = 3, P = 0.017), which is approximately one half of the effect obtained by anandamide alone (-23.3 ± 4.4%, n = 3, p = 0.03). This result suggests that TRPV1 and CB1 receptors contributed equally to the anandamide effect.

Adenosine 5'-triphosphate (ATP) is known to sensitize or potentiate CAP and heat evoked TRPV1 currents [7]. As shown in Figures 2A and 2C, the depression by CAP was blocked by the P_2_X and P_2_Y purinoceptor antagonist, suramin (100 μM). In addition, the depression by CAP was facilitated by the P_2_Y agonist, uridine 5'-triphosphate, in a dose-related manner (UTP, IC_50 _= 0.6 μM, Imax = -33%, N = 1.5) (Fig. 2B). We found that the CAP-dependent depression was significantly larger at 33°C, and the dose-response curve was shifted leaving the maximum effect unchanged (IC_50 _= 0.3 μM, Imax = -34%, N = 1.2) (Fig. 2B). Thus, we investigated the temperature dependent change of the excitation and its relation with TRPV1 channels.

### Action of temperature increase on net neuronal excitation

When the perfusate temperature was increased from 27°C to 33, 36 and 39°C, the magnitude of excitation evoked by the dorsal root stimulation was depressed (Fig. 3A). The effect was reversible, and was most significant in the superficial dorsal horn, as shown in Figure 3B, where the magnitudes of excitation at 27°C and 33°C are plotted along dorso-ventral lines. The optical response recorded at 36°C and 39°C often deteriorated gradually by the loss of slice viability. Therefore, we set the bath temperature at 27°C where stable recording over 5 hours could be achieved consistently, and increased the temperature when necessary. Figure 3C exhibits the magnitudes of the optical response in lamina II when the temperature was increased from 27°C to 33°C for a period of 40 min. The effect was significant (-19.7 ± 2.65%, n = 3, p = 0.009), and was reversible, although the excitation was somewhat augmented after recovery (5.86 ± 4.19%, n = 3, p = 0.5). The percent decrease of excitation was linearly related at -3%/°C for the temperature range of 27°C to 39°C over an average of four slices (Fig. 3C).

It is possible that the temperature dependent decrease of neuronal excitation observed in the superficial dorsal horn might be due to a property of the voltage-sensitive dyes used in the experiment. The following results, however, indicate that it is unlikely, and the observation is due to the temperature-dependent activation of TRPV1 channels, possibly by residual or released ligand(s).

As shown in Figure 4A, in the medium containing no Ca^++ ^ions, which are the primary ions flowing through TRPV1 channels, the temperature increase from 27°C to 33°C did not induce significant change in the magnitude of neuronal excitation evoked by the dorsal root stimulation (-4.73 ± 2.49%, n = 3, p = 0.74). Also, in the presence of the TRPV1 channel antagonist, CPZ (20 μM), the temperature increase from 27°C to 33°C did not affect the neuronal excitation (-7.13 ± 0.57%, n = 3, p = 0.18).

We further investigated whether ATP modulates the temperature-dependent change of the evoked excitation (Fig. 4B). The P_2_X and P_2_Y antagonist, suramin (100 μM, 15 min), did not affect the neuronal excitation at 27°C (1.43 ± 3.31%, n = 4, p = 0.70). In the presence of suramin, the temperature increase from 27°C to 33°C produced an insignificant change in excitation (-8.01 ± 2.70%, n = 4, p = 0.06). The P_2_X antagonist, 2',3'-O-(2,4,6-Trinitrophenyl) adenosine 5'-triphosphate (TNP-ATP, 1 μM, 15 min), was ineffective on the excitation at 27°C (102.40 ± 3.00%, n = 3, p = 0.50). In the presence of TNP-ATP, the excitation was suppressed by the temperature increase from 27°C to 33°C (-22.4 ± 2.80%, n = 3, p = 0.01).

GABA-and Glycine-containing inhibitory interneurons did not seem to contribute to the temperature dependency of excitation, because, in the presence of the inhibitory amino acid antagonists, BMI (10 μM) and STRY (3 μM), the excitation was depressed by the temperature increase significantly (-17.7 ± 0.43%, n = 3, p = 0.005) (Fig. 4C).

The voltage-gated Ca^++ ^channel blocker, Ni^++^, is known to reduce the synaptic transmission significantly. In the presence of Ni^++^(1 mM), the degrees of depression (-18.1 ± 0.97%, n = 4, p = 0.005) were identical to those observed in the control condition without Ni^++ ^(-19.7 ± 2.65%, n = 3, p = 0.0009) (Fig. 4C). The temperature increase also depressed the excitation recorded in the presence of the excitatory amino acid receptor antagonists, D(-)-2-Amino-5-phosphonovaleric acid (D-APV, 50 μM) and 6-Cyano-7-nitroquinoxaline-2,3-dione (CNQX, 10 μM), where presynaptic excitation predominates in the recorded optical response [14]. These results are consistent with the possibility that the presynaptic excitation is strongly temperature dependent, although the postsynaptic excitation could be also temperature dependent. These pharmacological properties of temperature effect are summarized in Figure 5.

### Presynaptic action of capsaicin and temperature

Since TRPV1 channels are abundant on presynaptic sites rather than on postsynaptic cells in the dorsal horn [12], we directly tested the CAP effect on the excitation of presynaptic elements. The presynaptic fibers and terminals were anterogradely stained with the voltage-sensitive dye that was applied by the suction pipette used for dorsal root stimulation [17]. Dorsal root stimulation of C fiber-activating strength elicited excitation most intensely in lamina II, as seen in Figure 6A. Since the response was altered very little by the perfusion of Ca^++^-free medium (e.g., Fig. 6B), we can conclude that only the presynaptic elements were stained and the excitation of presynaptic elements was recorded. A 10 minute application of CAP (1 μM) depressed the presynaptic excitation in lamina II significantly (-20.4 ± 6.54%, n = 5, p = 0.02) (Fig. 6A). CAP was ineffective in the Ca^++^-free medium (-2.09 ± 1.03%, n = 5) (Fig. 6B).

**Figure 7:**
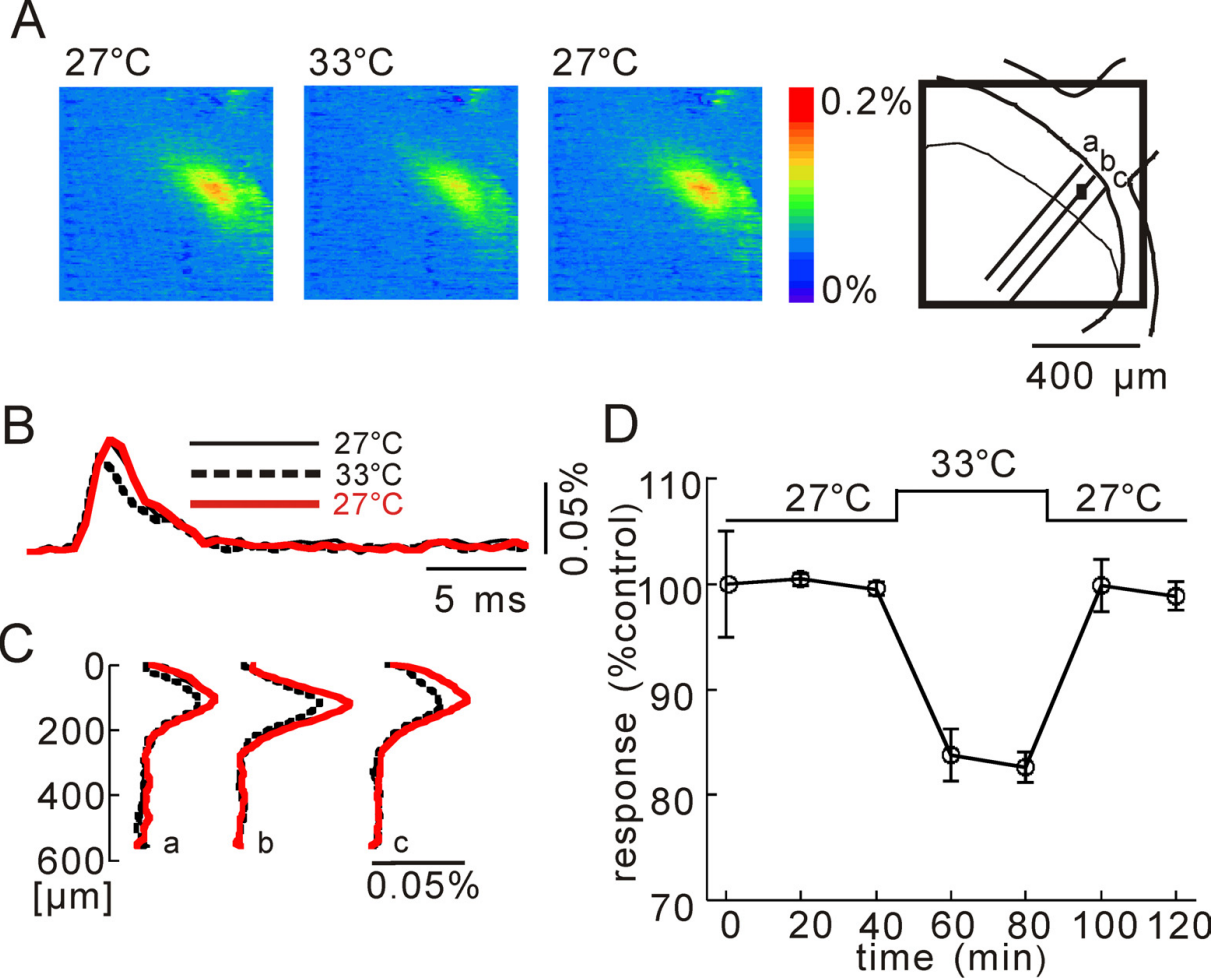
**Effect of temperature increase on presynaptic excitation**. **A**) Pseudo-color image sequence of stimulation-induced optical responses measured in the solution at 27°C, 33°C, and 27°C in a slice (from left to right). **B**) Superimposed time traces of optical signals obtained at 27°C (black), 33°C (dashed black), and 27°C (red). These records were obtained by spatial average of the optical signals of 8 × 8 pixels corresponding to a black square area (50 × 50 μm) shown in the schematic drawing of the dorsal horn. Dotted line in the optical signal records indicates the baseline level. **C**) Spatial distributions of the magnitude of excitation along dorso-ventral lines a, b and c in schematic drawing. **D**) Normalized time course of magnitude of optical signals showing the averaged effect induced by a change of temperature from 27°C to 33°C.

When the perfusate temperature was increased from 27°C to 33°C, the magnitude of presynaptic excitation was depressed (Fig. 7A and 7B). As seen in Figure 7C, the presynaptic excitation was largest in lamina II, and was depressed by the temperature increase. Figure 7D exhibits the time course of the change in magnitude in lamina II induced by a temperature increase from 27°C to 33°C for a period of 40 min. The effect observed in lamina II was significant (-17.3 ± 1.4%, n = 3, p = 0.006), and was reversible (1.2 ± 1.2%, n = 3, p = 0.42).

## Discussion

In the present study, we showed that CAP, anandamide and an increase in perfusate temperature inhibited the net excitation of pre and postsynaptic elements and the presynaptic excitation evoked by dorsal root stimulation in the superficial dorsal horn. These inhibitions were Ca^++^-dependent, antagonized by a TRPV1 channel antagonist and a P_2_X and P_2_Y antagonist, and facilitated by a P_2_Y agonist, but not by a P_2_X agonist. In addition, the inhibitions were unaffected by antagonists of GABA_A _and glycine receptors.

Several studies have shown that an intrathecal injection of CAP induces analgesic action at the spinal level [1-5]. In addition, electrophysiological studies have revealed that CAP suppresses C-fiber-evoked synaptic transmission in the spinal dorsal horn [19,20]. A bath application of CAP for 1 min produces a large increase in the spontaneous EPSCs frequency using patch clamp recording from spinal dorsal horn neurons [21]. However, the mechanism of these effects has not been clarified. In this study, we showed that CAP inhibits neuronal excitation, especially in superficial layers of the spinal dorsal horn. This distribution of inhibited area by CAP agrees with the distribution of TRPV1 channels reported by immunohistochemical studies [12]. It is well known that the activation of TRPV1 channels induces an influx of Ca^++ ^and Na^+ ^ions via this receptor [6]. In this study, we showed that inhibition of the neuronal excitation by CAP was not observed in the presence of CPZ. In addition, the CAP effect was not dependent on the activation of inhibitory amino acid-containing neurons, since this inhibition was unaffected by BMI and STRY. Therefore, it is reasonable to conclude that CAP inhibited the neuronal excitation via TRPV1 channels in superficial layer of the spinal dorsal horn, although a possibility remains that CAP-sensitive interneurons containing other inhibitory transmitter(s) and/or modulator(s) contributed to the CAP effect.

Next, we investigated the Ca^++ ^dependency of the CAP and temperature effects. Calcium ions are the major cation carried through the TRPV1 channel [6]. Both the CAPs- and temperature-dependent inhibitions were blocked in Ca^++^-free condition. Although the TRPV1 channel has a relatively high permeability to Ca^++^, both CAP and heat are known to activate the channels in the absence of external Ca^++ ^[6,22], and it seems difficult to explain the complete block of CAP and heat effects in the Ca^++ ^free condition. It is possible that TRPV1 channels were activated by CAP and heat in the Ca^++ ^free condition, but that the influx of cations other than Ca^++ ^via TRPV1 channels, and the following presynaptic depolarization, might not be sufficient to inhibit the neuronal excitation.

In the periphery, the acute administration of CAP induces burning pain by activating C fibers via TRPV1 channels. It depolarizes the terminals generating action potentials. They are conducted to the spinal cord and induce nociceptive sensation. Larger doses and/or prolonged administration of CAP have analgesic effect due to the desensitization of TRPV1 channels [23]. We recorded the excitation centrally. The depolarization of afferent terminals and/or fibers branching points would cause reduction of action potential size and even failure of action potential generation [26-29]. The central inhibitory action of CAP observed in this study accords well with this presynaptic inhibition mechanism. When CAP was applied for 10 min, the inhibition of neuronal excitation was reversible. However, when CAP was applied for 20 min, it became irreversible. This prolonged irreversible effect of CAP might be due to other mechanisms, such as CB1 activation.

In this study, the neuronal excitation was also inhibited by anandamide to a larger extent (-23.3%) than by CAP (-11.8%), and the effect of anandamide was partially antagonized by CPZ. It is known that the cannabinoid CB1 receptors are also activated by anandamide [30]. Therefore, it is possible that the inhibition of the neuronal excitation by anandamide is the combination of TRPV1 and CB1 channel activation.

TRPV1 channels in the periphery are activated by heat, as well as by CAP [6,7]. However, the temperature dependence of TRPV1 channels in the spinal dorsal horn has not been reported. In this study, we showed that increasing the perfusate temperature inhibited the neuronal excitation in the superficial laminae of the spinal dorsal horn. This inhibition was Ca^++^-dependent, antagonized by CPZ, and was unaffected by BMI and STRY, as well as, by the inhibition by CAP. From these results, the inhibition of neuronal excitation by increasing the perfusate temperature may be induced by the same mechanism as the inhibition induced by CAP. That is, the natural ligand of TRPV1 channels is present in the normal condition, and is continuously activating TRPV1 channels. In fact, in the natural condition, anandamide has been found in the dorsal horn [18], and we showed in this study that anandamide depressed the neuronal excitation in a similar manner to CAP.

Normally, the thermal threshold for activation of TRPV1 channels is more than 43°C [6,7]. However, in this study, the perfusate temperature increase from 27 to 33°C was sufficient to inhibit the neuronal excitation mediated by TRPV1 channels. Tominaga et al. [7] reported that the temperature threshold for TRPV1 channels activation was reduced from 42 to 35°C in the presence of ATP by measuring the current in HEK293 cells and that this effect was mediated by the P_2_Y receptor. In this study, the effect of CAP was blocked by suramin and was facilitated by UTP, and the effect of increasing the perfusate temperature was blocked by suramin, but not by TNP-ATP. These results indicate that the threshold for activation of TRPV1 channels in the spinal dorsal horn is decreased by the activation of the P_2_Y receptor. ATP has been reported to be a fast synaptic transmitter in the spinal dorsal horn [24,25]. ATP release from primary afferent terminals may contribute to the decrease of threshold for TRPV1 channels activation. The presence of ATP in the unstimulated condition is also highly likely. Therefore, TRPV1 channels in the spinal dorsal horn may be activated even at physiological temperatures.

In this study, the optical signal was recorded by anterograde staining of only primary afferents. Since this optical response was not decreased in a Ca^++^-free medium (Fig. 6B) or in D-AP5 and CNQX [17], it reflects exclusively the voltage change of presynaptic elements. The inhibition of the presynaptic response by CAP and by the increase in perfusate temperature suggests a decrease in the amplitude or number of presynaptic action potentials in presynaptic elements. TRPV1 channels exist, not only on primary afferent terminals, but also on postsynaptic neurons in the superficial dorsal horn [12] though not conclusive at present. Therefore, the decrease of presynaptic excitation may be due to a direct effect on TRPV1 channels in primary afferent terminals, or indirect effects mediated by any factor(s) released from TRPV1 channel-activated postsynaptic neurons. However, the latter possibility is less likely since the effect of CAP was observed in the presence of D-AP5 and CNQX, when most of the fast afferent signal transmission to the postsynaptic sites is blocked, although a possibility remains that the afferent stimulation we used activated CAP-sensitive glutamate-containing interneurons via transmitter(s) other than glutamate. Therefore, the inhibition of the presynaptic response by CAP and the increase in perfusate temperature is likely due to a decrease in the amplitude or number of presynaptic action potentials via the activation of TRPV1 channels.

In the spinal dorsal horn, the regulation of presynaptic spiking is known in the context of presynaptic inhibition. The depolarization of primary afferent terminals decreases the amplitude of action potentials and, thus, produces the presynaptic inhibition [27,28]. In addition, the silent presynaptic afferent terminals may become activated after their liberation from the shunting of the action potentials at primary afferent terminals [27,29,31-33]. In the superficial laminae of the dorsal horn, TRPV1 channels are abundant, especially at presynaptic elements of unmyelinated C-fiber origin. Therefore, CAP and the increase in perfusate temperature may inhibit neuronal excitation in the spinal dorsal horn via TRPV1 channels at primary afferent terminals.

TRPV1 channels exist not only at sensory neuron somata and primary afferent terminals, but also on axonal membranes [34]. Furthermore, it is reported that the application of TRPV1 channel agonists at afferent fiber bundles blocks action potential conduction [8-11]. Therefore, another possibility is that the inhibition of neuronal excitation in the spinal dorsal horn by CAP and an increase in perfusate temperature may be caused by **a **conduction block of the action potential in primary afferent fibers. It has been reported that removal of external Ca^++ ^does not affect the C fiber conduction block caused by CAP [9]. Therefore, the decrease of neuronal excitation by CAP and temperature increase is less likely due to a conduction block. It is difficult to distinguish, however, whether the effect of CAP and an increase in perfusate temperature is caused by a change of action potential at the level of primary afferent fibers or their terminals in the dorsal horn, because the presynaptic response that we measured in this study was the sum of excitations in both afferent fibers and terminals along the slice depth. To elucidate this point, it is necessary to record the action potentials in individual afferent fibers with higher-resolution camera in the future.

## Conclusion

The electrically evoked neuronal excitation in the spinal dorsal horn was prevented by CAP and by the increase in perfusate temperature via TRPV1 channels. These inhibitions were caused by the depression of primary afferent fiber terminal excitability, at least in part. Our results showed that these inhibitions were modulated by the P_2_Y receptor subtypes, and it may play a role to enable activation of TRPV1 channels at physiological temperature. This study clarifies the properties of TRPV1 channels in the spinal dorsal horn and the mechanism of analgesic effect by CAP at the spinal level. These findings may have clinical importance, especially for the control of pain.

## Methods

The preparation, apparatus, and data processing for the optical imaging were identical to those used in our previous studies [[13-16,35]; for detailed descriptions see [16]]. A brief summary follows.

### Preparation

All animal studies were undertaken with protocols approved by the university animal ethics committee. Transverse slices (350–450 μm thick) with dorsal roots attached (5–10 mm in length) were prepared from lumbosacral enlargements of 12- to 25-day-old Sprague-Dawley rat spinal cords, as described elsewhere [36]. A slice, stained with a voltage-sensitive absorption dye, RH-482 (0.1 mg/ml, 20 min), was set in a submersion-type chamber (0.2 ml) on an inverted microscope (IMT, Olympus, Tokyo) equipped with a 150-W halogen lamp. The slice was perfused with Ringer solution at 27°C containing 124 mM NaCl, 5 mM KCl, 1.2 mM KH_2_PO_4_, 1.3 mM MgSO_4_, 2.4 mM CaCl_2_, 26 mM NaHCO_3_, 0.2 mM thiourea, 0.2 mM ascorbic acid, and 10 mM glucose (oxygenated with 95% O_2 _and 5% CO_2_).

The RH-482 (NK-3630) dye was obtained from Nippon Kanko Shikiso (Okayama, Japan); the DL-2-amino-5-phosphonovaleric acid (D-AP5), bicuculline methiodide, strychnine hemisulfate, and nifedipine were from Sigma (St. Louis, MO); and the other chemicals were from Nacalai Tesque (Kyoto, Japan). Each of these chemicals was dissolved in distilled water at high concentration, divided into aliquots, and kept frozen at -40°C until use. The aliquots were dissolved in the bathing solution at known concentrations during the experiments. The perfusion solution of the slice was switched to the drug-containing solution for a fixed period.

### Neonatal capsaicin treatment

Capsaicin was dissolved in 100% ethanol, and then diluted with Tween80/PBS solution until ethanol concentration became 10%. Rats of postnatal day 2–3 were injected the capsaicin solution (50 mg/kg) subcutaneously at the dorsal cervix. Three weeks after the injection, rats were tested on a hot plate (65°C). While normal untreated rats raised and licked the feet within 10 seconds, successfully treated rats did not react over one minute.

### Optical recording

The light absorption change in a 0.83-mm-square area in the dorsal horn at a wavelength of 700 ± 32 nm was recorded by an imaging system (SD1001, Fuji Film Microdevice, Tokyo) with 128 × 128-pixel photo sensors at a frame rate of 0.6 ms [37]. Thirty-two single pulses were given to the dorsal root at a constant interval of 12–15 s. Starting at 10 ms before each stimulus, 128 consecutive frames of the light-absorption images were taken by the image sensor with a sampling interval of 0.6 ms. The reference frame, which was taken immediately before each series of 128 frames, was subtracted from each of the subsequent 128 frames. Thirty-two series of such difference images were averaged and stored in the system memory. We determined the initial frame by averaging the first 15 frames of the difference image and then subtracting this from each of the 128 frames of the image data on a pixel-by-pixel basis in order to eliminate the effects of noise contained in the reference frame. The ratio image was then calculated by dividing the image data by the reference frame. In most cases, the ratio image was filtered by a 3-point moving average over time [see [14] for detail].

### Dorsal root stimulation

The dorsal root was stimulated by a glass suction electrode. The types of primary afferent fibers activated by the electrical stimuli were initially identified by the field potentials recorded by a glass microelectrode positioned either in the superficial dorsal horn or at the entry zone of the root, as described previously [14].

The single current-pulse stimulation of the dorsal root elicits the following optical responses in the dorsal horn [14]: (1) a brief (< 3 ms) and small, almost undetectable, response is evoked at the entry zone of the dorsal root and, occasionally, in the deep dorsal horn by a 0.05-mA current pulse of 0.05 ms duration; (2) by increasing the stimulus intensity to 0.1 mA, an optical response of longer duration (< 100 ms) appears in lamina I extending to the outer part of lamina II, and in lamina III and deeper laminae; (3) additional increases in intensity (> 0.3 mA) and/or duration (> 0.5 ms) lead to the generation of an intense, prolonged (> 200 ms) response in the superficial laminae I-III, most prominently in lamina II. The long-lasting response in lamina II is delayed, with the latency corresponding to the slow conduction velocity of C fibers (ca. 1 m/s). The onset of optical responses in lamina I and lamina III or deeper laminae elicited by any of these conditions takes place within one image frame (i.e., the latency is less than 0.6 ms). The conduction velocity of fibers responsible for the induction of the immediate response should be faster than 6 m/s (dorsal root length of 4 mm/0.6 ms), which corresponds to the conduction velocity of A fibers. These spatial and temporal profiles of optical responses agree fairly well with the cytoarchitectonic organization of the dorsal horn [38-41], giving additional indications of the fiber types activated by the stimuli.

Therefore, **t**he activation conditions consisted of a 0.05-mA current pulse of 0.05 ms duration for Aα/β fibers (A fib**e**r other than Aδ fibers), a 0.1-mA current pulse of 0.05 ms duration (low-intensity stimulation) for Aα/β/δ fibers (all types of A fibers), and a 1.5-mA current pulse of 0.5 ms duration (high-intensity stimulation) for A and C fibers. These parameters were similar to those used in other studies [42-46].

### Temperature control

The temperature of perfusate in the recording chamber was maintained with a heating coil along the polyethylene tubing (50 cm in length and 1 mm in diameter) between a peristalic pump and the inlet of chamber. Solution in a reservoir that was maintained at a room temperature of 25°C was heated to 27°C while passing through the tubing. The solution temperature in the chamber was continuously monitored with a small sensor positioned near the specimen. Since the room temperature was maintained constant and the solution in the reservoir was equilibrated to the room, the solution temperature in the chamber was usually stable, within ± 0.2°C, during the duration of each experiment (1–3 hours). Since the capacity of the chamber (0.5 ml) and the volume in the tubing (0.1 ml) were small in respect to the flow (3 ml/min), the temperature of the solution in the chamber could be controlled by the amount of electric current. In control experiments, the temperature measured at the surface of the slice was increased from 27°C to 33°C within 180 seconds by a current increase, and, following the reduction after 20 min at 33°C, the temperature at the surface of the slice was returned to 27°C within 300 seconds.

### Statistical analysis

Results were expressed as the mean ± SE. Non-parametric ANOVA (Tukey-Kramer test) was used for statistical difference**s**. Significant differences were calculated using the t-test, and differences were considered significant when p < 0.05 (*) or p < 0.01 (**) and # (p > 0.5).
